# Aromatic and proteomic analyses corroborate the distinction between Mediterranean landraces and modern varieties of durum wheat

**DOI:** 10.1038/srep34619

**Published:** 2016-10-06

**Authors:** Federico Vita, Cosimo Taiti, Antonio Pompeiano, Zuguang Gu, Emilio Lo Presti, Larisa Whitney, Michele Monti, Giuseppe Di Miceli, Dario Giambalvo, Paolo Ruisi, Lorenzo Guglielminetti, Stefano Mancuso

**Affiliations:** 1 Department of Agriculture, Food and Environment, University of Pisa, Via Mariscoglio 34, 56124, Pisa, Italy; 2 LINV-Department of Plant Soil and Environmental Science, University of Florence, Viale delle idee 30, I-50019 Sesto-Fiorentino (FI), Florence, Italy; 3 Laboratory of Ecological Plant Physiology, Global Change Research Institute CAS, Bělidla 986/4a, CZ-603 00 Brno, Czech Republic; 4 Division of Theoretical Bioinformatics (B080), German Cancer Research Center (DKFZ), Im Neuenheimer Feld 280, 69120 Heidelberg, Germany; 5 Heidelberg Center for Personalized Oncology (DKFZ-HIPO), German Cancer Research Center (DKFZ), Im Neuenheimer Feld 280, 69120 Heidelberg, Germany; 6 Department of Agricultural Sciences, Mediterranean University of Reggio Calabria, Salita Melissari, 89124 Reggio Calabria, Italy; 7 DISVA, Department of Life and Environmental Science, Marche Polytechnic University, Via Brecce Bianche, 60131, Ancona, Italy; 8 Department of Agricultural and Forest Sciences, University of Palermo, Viale delle Scienze, Ed. 4, 90128 Palermo, Italy; 9 Fondazione A. e S. Lima Mancuso, Piazza Marina, 61-90133 Palermo, Italy

## Abstract

In this paper volatile organic compounds (VOCs) from durum wheat cultivars and landraces were analyzed using PTR-TOF-MS. The aim was to characterize the VOC’s profile of the wholemeal flour and of the kernel to find out if any VOCs were specific to varieties and sample matrices. The VOC data is accompanied by SDS-PAGE analyses of the storage proteins (gliadins and glutenins). Statistical analyses was carried out both on the signals obtained by MS and on the protein profiles. The difference between the VOC profile of two cultivars or two preparations of the same sample - matrices, in this case kernel vs wholemeal flour - can be very subtle; the high resolution of PTR-TOF-MS - down to levels as low as pptv - made it possible to recognize these differences. The effects of grinding on the VOC profiles were analyzed using SIMPER and Tanglegram statistical methods. Our results show that it is possible describe samples using VOC profiles and protein data.

Wheat is the most cultivated crop in temperate regions, and is widely used for human food and livestock feed^1^. The global production of wheat (2015) was 733 MT (http://www.fao.org/worldfoodsituation/csdb/en/). The two most popular species are “bread” or spring wheat (*Triticum aestivum* L.) and “pasta” or durum wheat *(Triticum durum* Desf.) that occur as natural intergeneric hybrids^2^. About 95% of the wheat currently grown worldwide is hexaploid spring wheat; it is sown in almost every agricultural region of the globe^1^. The remaining 5% of the world production is the tetraploid durum wheat, though within the Mediterranean basin it is one of the leading food crops where it is the primary cereal grain used in the production of pasta and certain bread^3^,^4^ products. Italy is the world’s top producer of pasta and has the highest pro capita consumption rate (http://www.internationalpasta.org). In Italy, given its economic importance, durum wheat has long been a focus of breeding programs, from which numerous cultivars have derived. Many of these cultivars are currently grown both inside and outside the country. Historically in Italy, we can distinguish three main periods in breeding trends in the 20^th^ and 21^th^ century. Landraces and genealogical selections (‘old’ cultivars) characterized the pre-1950 era. Starting around 1950 and for the next quarter century or so, new mutagenesis^5^ and crossing techniques were added to the effort to obtain cultivars with improved yield (‘intermediate’ cultivars). Cultivars developed post-1974 (‘modern’ cultivars) contain *Rht* genes which created shorter plants and thus reduced lodging, but also modified the properties of the gluten in a way that was favorable for the texture of bread and pasta products^6^. These modern cultures have consequently displaced the older cultivars and landraces^7^,^8^.

In modern cultivars, improvements in yield and is most often measured in terms of the response to different nitrogen availabilities and varying thermo-photoperiodicity before and after anthesis^5^. Genetic erosion resulting from the displacement of landraces is a significant problem in current breeding programs^7^. It is likely that the old cultivars are the most valuable source of genetic diversity and consequently these may hold the potential introducing many desirable traits into the modern cultivars. Indeed, this is the case for some Mediterranean durum landraces^9^. Therefore, the local landraces could potentially provide a source of new alleles for the improvement of commercial value^7^. The introgression of these alleles that allow for growth in suboptimal environments is currently of particular interest^10^. Traits of interest could include resilience of plants faced with adverse conditions such as pests attacks, diseases, adaptability to low-input farming, and abiotic stressors^11^,^12^. Although the way quality is defined does to an extend depend on the intermediary user and the intended end-use^13^, at least two studies have shown how increased genetic variability may correlate with traits often associated with quality^14^,^15^. Gluten strength is a key factor used to define durum wheat quality^16^. The genotype of a cultivar can influence gluten strengh by determining the quantity of gluten proteins which accumulate in the grain, and also quality - as defined by the specific alleles present. Gluten proteins are classified into gliadins and glutenins based on their different solubility in aqueous alcohols^17^,^18^. Monomeric gluten proteins show viscous behavior, whereas polymeric gluten proteins (glutenins) are elastic^19^. There are four types of gliadins:, α-, β-, γ-, and ω-. The glutenin subunits are grouped into high molecular weight (HMW-GS) and low molecular weight (LMW-GS)^20^. The amount of high and low weight subunits determines gluten strength^21^,^22^.

Qualitative and quantitative differences in gliadin and glutenin composition are typically used for discriminating wheat cultivars. A variety of techniques have been used to characterize these proteins including gel electrophoresis^23^, capillary electrophoresis (CE)^24^, Reverse Phase HPLC (RP-HPLC)^25^, two-dimensional electrophoresis (2DE) coupled with nano-liquid chromatography/electrospray ionization ion trap-mass spectrometry/mass spectrometry (nano-HPLC−ESI-IT-MS/MS)^26^ and matrix-assisted laser desorption/ionization time-of-flight mass spectrometry (MALDI-TOF-MS) mass spectrometer^27^. While these methodologies have certainly made a notable contribution to the characterization of varieties, additional methods that examine specific features may also be used.

The analysis of aromatic profiles constitutes a newer approach to characterizing wheat varieties and landraces. Volatile organic compound (VOC) emissions have been used to identify varieties and landraces in cooked grains of wheat^28^ and to describe the volatile composition of durum wheat semolina and pasta^29^. Although these products aren’t usually renowned for their aromatic properties^29^, new evidence suggests that the VOC profile of a flour may to some degree predict the aroma of the processed end products^30^,^31^. Also, VOCs from the grain can be used to differentiate cereal species^32^ and VOCs have successfully been used to differentiate among wheat varieties (analysis done on cooked grain)^33^. Wholemeal flour exhibits concentrations of VOC components that are different from milled white flour^34^. This implies that wheat flour constitutes an important source of odorants can influence the aroma of finished bread products. It is common that compounds that contribute to aroma be present in trace or ultra-trace amounts, and that the final aroma be comprised of numerous classes of chemical compounds^35^. VOC analysis in wheat can be done - for example - via gas chromatography-mass spectrometry (GC-MS) coupled with suitable techniques to concentrate volatiles such as dynamic headspace extraction^33^ or via the headspace solid-phase microextraction^29^ (HS-SPME). The benefits of using GC-MS based methods rather than other analytical systems have been investigated. One such other system is the “Proton Transfer Reaction-Mass Spectrometer (PTR-MS)”, a soft chemical ionization procedure that allows real time visualization of the flow of VOCs contemporary to the moment their signal is picked up by the detector present in the instrument. This allows for the elimination of the inconvenient delay associated with mass spectrometry/gas chromatography dependent methods, thus allowing for a massive increase in through-put potentiality. This is undoubtedly a powerful new instrument considering that it also has a limit of detection that allows reliable identification of compounds present in concentrations as low as a few pptv (parts per trillion by volume)^36^. Further improvements have been made to PTR-MS technology by coupling it with time-of-flight (TOF-MS). PTR-TOF-MS instruments can generate entire mass spectra (snapshots) of complex gas mixtures in short response times with high mass resolution and with virtually no upper mass limit^37^.

Taking full advantage of the latest improvements in the analytical technology, this study aims to describe the VOC profile of 47 wheat varieties and landraces (44 of durum wheat) by analyzing the kernels and the resulting wholemeal flour and to confirm the presence of specific volatiles profiles through statistical analyses. The effect of the mechanical grinding was then evaluated by comparing kernel data with data from wholemeal flour. Finally, the kernel storage proteins (gliadins, glutenins) were analyzed by SDS-PAGE in order to complete the description of the samples.

## Results

### VOC analysis by PTR-TOF-MS

PTR-TOF-MS analysis of the kernel and wholemeal flour of 47 wheat varieties (Table 1) led to the identification of a total of 32 compounds (Table 2). The majority of these (26 out of 32) were observed in the range of 30-100 *m/z*.

**Figure 1:**
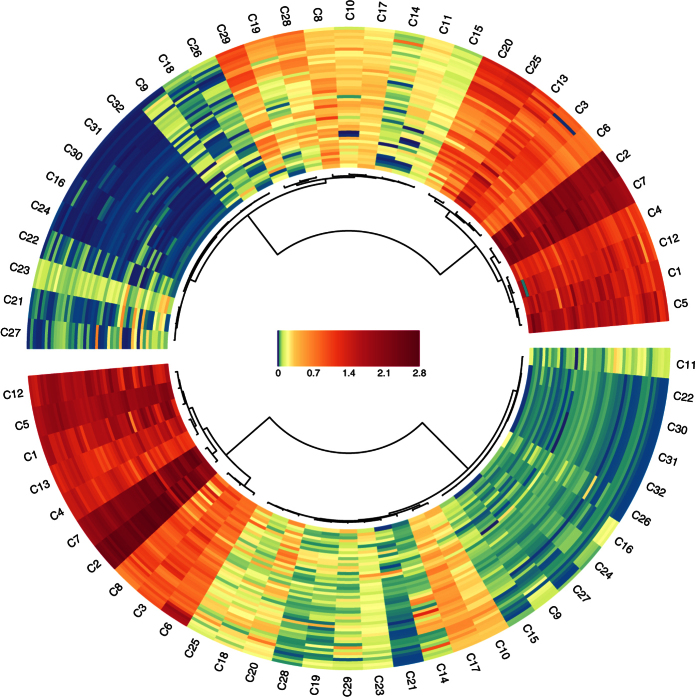
(*Lower panel*) Circular heat map representation of unsupervised hierarchical clustering of the data coming from analysis of 47 *kernel* samples (rows) grouped by compound type (columns); (*Upper panel*) Circular heat map representation of unsupervised hierarchical clustering of the data coming from analysis of 47 *wholemeal flour* samples (rows) grouped by compound type (columns). Shades of red and blue represent increase and decrease of a compound relative to the median compound levels (see color scale).

The quantitative data are depicted in the form of a circular heat map (Fig. 1). For each compound (wedges), the amount present in the 47 samples (lines) is compared to how much the other compounds are present. The figure can be read as follows: Firstly, the absolute concentration of a specific compound - the “average” for all samples - is depicted by the overall colour of the wedge. A second important aspect described by the figure is how the concentration of that compound varies within the samples. This is visible by how homogeneous the colour of the wedge is. The analysis of these two aspects combined is referred to as the analysis of “compound trends”. A dendrogram was constructed based these compound trends using the correlation distance and Ward’s method^38^ and it is positioned in the internal part of the circle.

### VOC analysis of kernels

It is clear that the overall amount of VOC varies greatly from compound to compound (Fig. 1, lower half). Also, a single compound may be present in large amounts in some species but very small amounts in others. This is the case for example for dimethyl sulphide (C14, C_2_H_6_S) and diethyl sulphide (C25, C_4_H_10_S), which contain within their respective wedges both bright red and blue lines - colours which are on the opposite end of the scale. Nonetheless, in most cases, there is at least some degree of homogeneity, meaning that if a single compound is present in relatively low amounts in one sample, it is usually is present in the others on the lower end of the scale as well.

We can observe how the dendogram splits the samples into two major branches (Fig. 1, lower half). One branch goes to the compounds which are present in the largest quantities and are prevalently those with low molecular weight. The top two compounds in terms of quantity are methanol (C2, CH_4_O) and acetaldehyde (C7, C_2_H_4_O). For the most part, the high molecular weight molecules are present in lower quantities and usually are found under the other main branch of the heat map dendrogram. Within this second branch, three compounds stand out, as their overall quantity is greater than the other compounds present in that branch and tending towards concentrations more typical of the first branch: 2-propenal (C10, C_3_H_4_O), 2-butanone (C17, C_4_H_8_O) and benzene (C20, C_6_H_6_).

**Figure 2:**
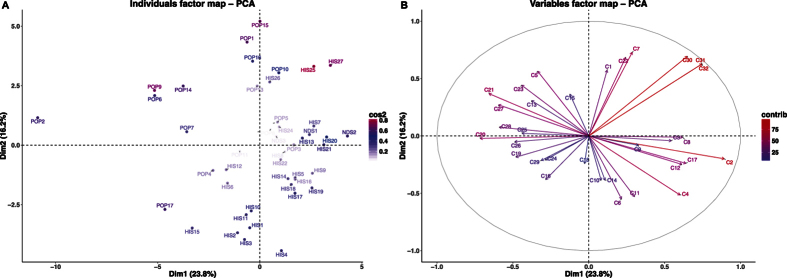
(**A**) Principal component analysis (PCA) results of PTR-TOF-MS data obtained from 47 durum wheat *kernel* samples. Dim1 = first dimension (PC1), Dim2 = second dimension (PC2); (**B**) Vector representation of the contribution of each compound to the distinction of *kernel* samples. The length of the vectors is directly correlated to their significance within each population. Between vectors and between a vector and an axis, there is positive correlation if the angle is less than 90 degrees whereas the correlation is negative if the angle reaches 180 degrees. There is no linear dependence if the angle is 90 degrees.

The PCA (Fig. 2A) performed on kernel data showed that the first two dimensions (PC1 and PC2) account for 40.0% of the total variance (total inertia). The first axis (PC1) explains 23.8% of the total variance and the second axis (PC2) 16.2%. From the spatial distribution of samples we observe that Claudio (POP2) distinguishes itself the most. Other modern varieties like Duilio (POP6), Dylan (POP7), Iride (POP 9) and Simeto (POP14) form a group separate from other accessions, all (including POP2) in the upper left quadrant of the PCA.

The correlation circle (Fig. 2B) of the samples was constructed based on specific compounds that associated with the first axis of the PCA (Table 3). The compounds that positively correlate with the first axis of the PCA (PC1) are mainly methanol (C2, CH_4_O), 2-phenylethyl alcohol (C32, C_8_H_10_O), acetophenone (C31, C_8_H_8_O) and 2,3-dimethylpyrazine (C30, C_6_H_8_N_2_) whereas benzene (C20, C_6_H_6_), the terpene fragment (C21, C_6_H_8_), methylsulphanylmethane (C27, C_2_H_5_O_2_S) and phenylethene (C28, C_8_H_8_) correlate negatively.

The following compounds positively correlate with the second dimension of the PCA: acetaldehyde (C7, C_2_H_4_O), and C6 fragment (C22, C_6_H_10_). Two alkyl fragment compounds (C6, C_3_H_6_; C11, C_4_H_8_) negatively correlate with PC2. In this dataset, most of the landraces grouped according to PC2 in the third and fourth quadrants: Aziziah (HIS1), Biancolilla (HIS2), Biancuccia (HIS3), Bidì (HIS4), Dauno (HIS10) and Dauno III (HIS11).

The contribution of individual compounds to sample differentiation was displayed as a correlation circle (Fig. 2B) where normalized vectors graphically represent the quantitative variables. The length and the direction of the vectors directly correlate with their significance within each population. A positive correlation between compounds is greater when the angle between their directions is smaller (close to 0 degree), whereas the correlation is negative if the angle reaches 180 degrees. No linear dependence exists if the angle is exactly 90 degrees. In the kernel matrix, a clear positive correlation was identified among 2,3-dimethylpyrazine (C30, C_6_H_8_N_2_), acetophenone (C31, C_8_H_8_O) and 2-phenylethyl alcohol (C32, C_8_H_10_O).

### VOC analysis on wholemeal flour

The volatiles from wholemeal flour are depicted as a circular heat map (Fig. 1, upper half). Some of the compounds such as benzene (C20, C_6_H_6_) and diethyl sulphide (C25, C_4_H_10_S) seem to go hand in hand with each other in that they occur in very similar concentrations, just they did in the kernel matrix described above, and they increase by the same amount following grinding of the kernel into wholemeal flour. The heat map also displays some quantitative compound trends that do not correspond to those previously reported for kernel data. For example as phenylethene (C28, C_8_H_8_) and 1,3-dimethylbenzene (C29, C_8_H_10_). These two aforementioned compounds clustered in the first branch of the dendrogram, whereas the second branch contains only low molecular weight compounds like methanol (C2, CH_4_O) and acetaldehyde (C7, C_2_H_4_O).

**Figure 3:**
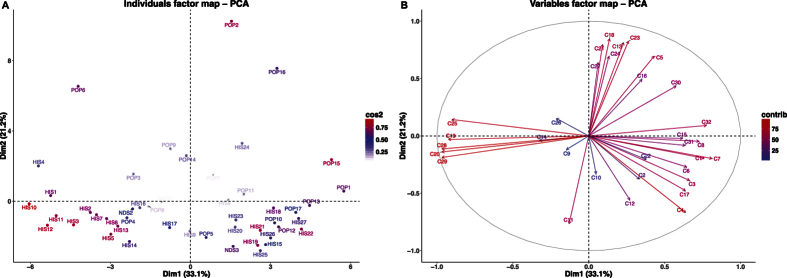
(**A**) Principal component analysis (PCA) results of PTR-TOF-MS data obtained from 47 durum wheat *wholemeal flour* samples. Dim1 = first dimension (PC1), Dim2 = second dimension (PC2); **(B)** Vector representation of the contribution of each compound to the distinction of *wholemeal flour* samples. The length of the vectors is directly correlated to their significance within each population. Between vectors and between a vector and an axis, there is positive correlation if the angle is less than 90 degrees whereas the correlation is negative if the angle reaches 180 degrees. There is no linear dependence if the angle is 90 degrees.

From the PCA analysis done on wholemeal flour data (Fig. 3A) we can see that the total variance along the two axes (PC1 and PC2) accounted for 54%, a considerable increase from the 40% it was in the PCA done on kernel data. PC1 accounted for 33.1% of the total variance whereas PC2 accounted for 21.2%. In this multi-dimensional space, modern varieties like Claudio (POP2), Duilio (POP6) and Iride (POP9) Tirex (POP16) separated. As to the distribution of the local landraces, Aziziah (HIS1) and Bidì (HIS4) are the only ones found in the upper left hand quadrant. Indeed, all other HIS varieties (with the exception of HIS24 in the upper right quadrant) are located in the lower part of the PCA.

In the correlation circle (Fig. 3B, Table 3), we notice that acetaldehyde (C7, C_2_H_4_O), 2-phenylethyl alcohol (C32, C_8_H_10_O), and acetylene (C1, C_2_H_2_) positively correlate with PC1, while diethyl sulphide (C25, C_4_H_10_S), 1,3-dimethylbenzene (C29, C_8_H_10_), phenylethene (C28, C_8_H_8_), propanethiol (C19, C_3_H_8_S) and benzene (C20, C_6_H_6_) negatively correlate. Compounds that positively correlate with PC2 include methyl acetate (C18, C_3_H_6_O_2_) and 2,3-butanedione (C23, C_4_H_6_O_2_) and two compounds related to alkyl fragments (C11, C_4_H_8_; C4, C_3_H_4_) negatively correlate.

In the correlation circle for wholemeal flour (Fig. 3B) 2-phenylethyl alcohol (C32, C_8_H_10_O) positively correlates with acetophenone (C31, C_8_H_8_O) and 2,3-dimethylpyrazine (C30, C_6_H_8_N_2_), although especially for the latter two compounds, this was not as nearly as strong as it was in the kernel results. In wholemeal flour, it was benzene (C20, C_6_H_6_), phenylethene (C28, C_8_H_8_) and 1,3-dimethylbenzene (C29, C_8_H_10_) which proved the most significant based on vector length. These latter three compounds positively correlated with each other, but they negatively correlated with the aforementioned C32 compound.

### Simper analyses

The PTR-TOF data was used in a further analysis known as SIMPER (Table 4). The objective of this analysis was to find key compounds that allow for differentiation of one sample from another when confronted in a pair-wise analysis. The results display the contribution of each compound to the average overall dissimilarity of the two confronted samples. A cut-off is imposed when ∑δi% reaches 70%.

The first analysis (Table 4A) confronted kernel and wholemeal flour data within the same species, and in this table the results from one species from each category (historical “HIS5”, not durum species “NDS1”, and modern “POP2”) are displayed. In this analysis, three compounds were statistically significant in each of the three categories: the isoprene fragment (C3, C_3_H_2_), ethanol (C8, C_2_H_6_O) and diethyl sulphide (C25, C_4_H_10_S).

SIMPER analysis was also used to confront kernel vs kernel and wholemeal flour vs wholemeal flour (Table 4B and 4C respectively) in the following comparisons: HIS5 vs NDS1, HIS5 vs POP2 and NDS1 vs POP2; in other words in the same matrix was used to confront different genotypes.

In the kernel vs kernel analysis, the compounds that were consistently present in all the three comparisons are represented by low molecular weight compounds like acetylene (C1, C_2_H_2_), methanol (C2, CH_4_O), alkyl fragments (C4, C_3_H_4_; C6, C_3_H_6_), acetaldehyde (C7, C_2_H_4_O), ethanol (C8, C_2_H_6_O), acetone (C12, C_3_H_6_O).

A key feature that distinguishes the wholemeal flour vs wholemeal flour analysis from the kernel vs kernel analysis discussed above, is the presence of high molecular weight compounds which, in the case of wholemeal flour vs wholemeal flour, turn out to be relevant (Table 4C). Such compounds are present in each of the three pair-wise comparisons among the compounds that contributed the most to overall dissimilarity. These compounds include benzene (C20, C_6_H_6_), diethyl sulphide (C25, C_4_H_10_S) and 1,3-dimethylbenzene (C29, C_8_H_10_), and they accompany the low molecular weight compounds acetylene (C1, C_2_H_2_), alkyl fragment (C4, C_3_H_4_), acetone (C12, C_3_H_6_O) and acetate (C13, C_2_H_4_O_2_). Acetate (C13, C_2_H_4_O_2_) is the only one out of these four low weigh compounds not reported in kernel results, whereas diethyl sulphide (C25, C_4_H_10_S) was the only one previously reported in the pair-wise comparison between matrices (Table 4A).

Considering the overall role of compounds in the discrimination of the selected samples (Bidì, HIS4; Khorasan, NDS1; Claudio POP2) we notice how specific compounds are consistently present in each of the three comparisons. SIMPER results that included Bidì (HIS4), showed that two compounds, the alkyl fragments (C4, C_3_H_4_) and acetone (C12, C_3_H_6_O), are statistically significant. For Khorasan (NDS1) the results highlighted the presence of acetone (C12, C_3_H_6_O) whereas Claudio (POP2) results shown the presence of a large amount of compounds. These are represented by small compounds like acetylene (C1, C_2_H_2_), methanol (C2, CH_4_O) and others characterized by a higher molecular weight like the terpene fragment (C21, C_6_H_8_) and diethyl sulphide (C25, C_4_H_10_S).

### Tanglegram

**Figure 4:**
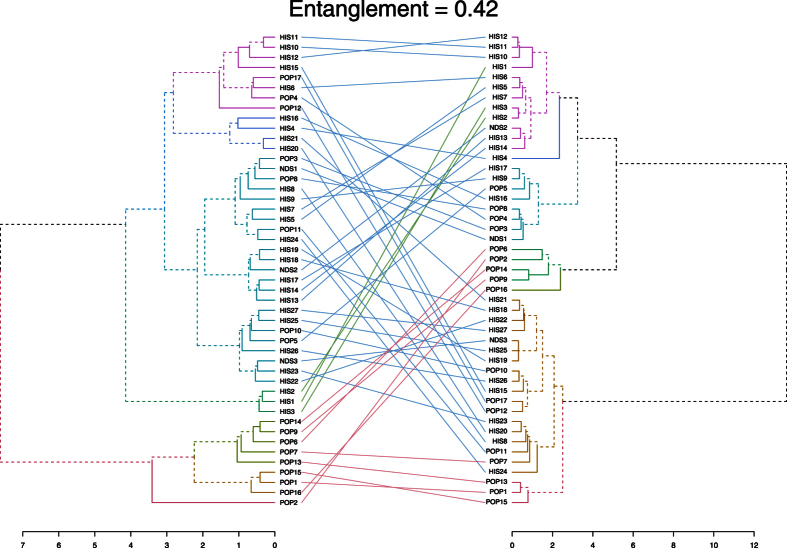
Tanglegram showing phylogenetic trees for the kernel (left tree) and wholemeal flour (right tree) from wheat samples. The linking lines connect the kernel and wholemeal flour data of the same sample. Entanglement is measured by giving the left tree’s labels the values of 1 till tree size, and then match these numbers with the right tree. Therefore, entanglement value is a measure between 1 (full entanglement) and 0 (no entanglement). A lower entanglement coefficient corresponds to a better alignment.

A tanglegram was constructed using quantitative VOC data from the kernel and wholemeal flour, to study the relationships among accessions (Fig. 4). The tanglegram is presented as two rooted phylogenetic trees that are linked according to quantitative trends within each sample. The entanglement value (0.42) indicates that phylogenetic trees are partially stackable, and from this we conclude that that the mechanical grinding strongly affected the VOC profile and thus the relationships among samples. The tree made from kernel data is on the left and the tree made form wholemeal flour data is on the right. Both trees were created according to the Ward method of agglomeration^38^.

Samples in the kernel tree (Fig. 4, left) grouped into two main branches. At a more detailed level though, the accessions grouped into seven clusters, each denoted by a different color. Starting from the top, the first of these such clusters (violet) is made up of samples belonging to both the HIS group and the POP group, with five and three accessions respectively. Moving down the tree, the next cluster, dark blue, is made up of exclusively of HIS samples: Bidì (HIS4), Margherito (HIS16) and two Russello samples. The medium blue cluster was the most populated group (23 accessions), with a prevalence of HIS landraces (15), all the NDSs (Khorasan, Maiorcone, Romano) and 5 modern varieties (Colombo (POP3), Creso (POP5), Hathor (POP8), Neolatino (POP10) and Normanno (POP11)). The dark green cluster grouped only HIS samples like Aziziah (HIS1), Biancolilla (HIS2) and Biancuccia (HIS3). The last three clusters (green, brown, and red) included only modern varieties, with Claudio (POP2) clustering separately from the rest of the samples.

Regarding the wholemeal flour phylogenetic tree (Fig. 4, right), we observe that the violet cluster is made up of HIS samples, including three samples that had clustered separately in kernel tree: Aziziah (HIS1), Biancolilla (HIS2), and Biancuccia (HIS3), as shown by the green connection lines. In this trend, only Maiorcone (NDS2), stands apart. It is a *Triticum aestivum* L. landrace. Moving down the tree, Bidì (HIS4), in dark blue clusters alone. The next cluster, medium blue, is made up of heterogeneous samples belonging to each of the three groups. The dark green cluster includes only POP samples, as previously observed in the kernel tree. The light green cluster was another single sample cluster, made up only of Tirex (POP16).

In the wholemeal flour tree, the brown cluster is the largest group (18 entries). In it, the majority of samples belong to the HIS group. The last cluster, red, grouped only three modern varieties: Aureo (POP1), Sculptur (POP13) and Svevo (POP15). These three samples didn’t form a separate cluster in the kernel tree, as shown by the tanglegram connections.

### SDS-PAGE results

In all 47 wheat varieties and landraces, gliadin and glutenin were analyzed separately. The aim was to verify the presence of specific protein profiles. The wholemeal flour matrix was used for all protein analyses.

### Glutenins

**Figure 5:**
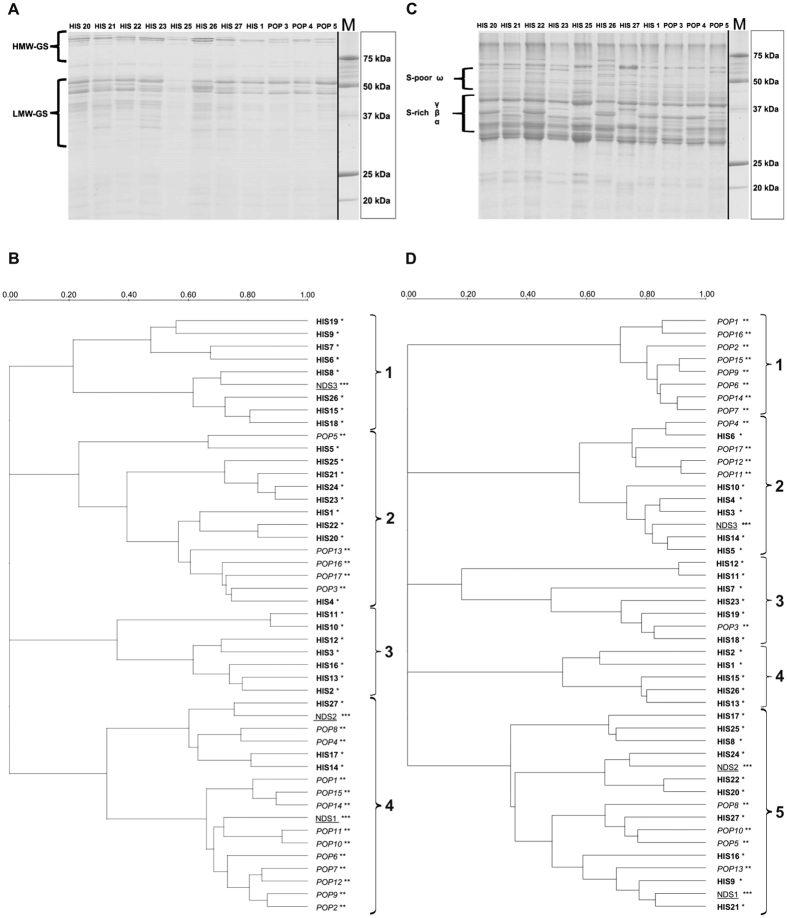
(**A**) SDS–PAGE analysis of reduced *glutenin* fractions from 12 samples. Glutenin classes were displayed on the left. Precision Plus Protein™ Unstained (Bio-Rad Laboratories, Inc, Hercules CA) was used as molecular marker. HMW: high molecular weight; LMW: low molecular weight; **(B)** Phylogenetic tree resulting from the analysis of *glutenin* fractions obtained by SDS-PAGE analysis. Images were processed with Quantity One software (Bio-Rad Laboratories, Inc, Hercules CA) using Ward’s method^38^ for clustering. For sample references see Table 1. The scale represents the percentage of similarity by the Dice coefficient; (**C**) SDS–PAGE analysis of reduced *gliadin* fractions from 12 samples. Gliadin classes were displayed on the left. Precision Plus Protein™ Unstained (Bio-Rad Laboratories, Inc, Hercules CA) was used as molecular marker; **(D**) Phylogenetic tree resulting from the analysis of *gliadin* fractions obtained by SDS-PAGE analysis. Image data were processed with Quantity One software (Bio-Rad Laboratories, Inc, Hercules CA) using Ward’s method^38^ for clustering. For sample references see Table 1. The scale represents the percentage of similarity by the Dice coefficient.

SDS-PAGE on glutenin fractions (Fig. 5A) allowed for the identification of specific profiles related to either HMW (high molecular weight) or LMW (low molecular weight) fractions. To graphically display the relationship among the samples, gel images were used to generate phylogenetic trees based on similarity comparison (Fig. 5B).

The glutenins grouped in 4 main clusters (Fig. 5B). With the exception of Romano (NDS3), the first cluster from the top includes only HIS samples: Bufala rossa (HIS6), Capeiti (HIS7), Cappelli (HIS8), Cicireddu (HIS9), Madonie (HIS15), Realforte bianco (HIS18), Realforte rosso (HIS19) and Sicilia (HIS26).

The second cluster showed the highest degree of diversity since it includes samples belonging to HIS - including all the Russello accessions (HIS20, HIS21, HIS22, HIS23 and HIS24) and POP (Colombo, POP3; Creso, POP5; Tirex, POP13; Sculptur, POP16; Valbelice, POP17). Within cluster 2, Aziziah (HIS1) clusters separately from other samples.

The third cluster contains only samples belonging to ancient landraces like Biancolilla (HIS2), Biancuccia (HIS3), Garigliano (HIS12), Gigante (HIS13), Margherito (HIS16). Within cluster 3, Dauno (HIS10) and Dauno III (HIS11), showed the highest degree of similarity to each other (>0.70 of DICE coefficient), with respect to their glutenin profiles. The last cluster (4) was the largest (17 samples) and could be divided in two sub-groups. The first comprising 6 samples contained a mixed composition with three HIS accessions, (Grifoni 235, HIS14; Perciasacchi, HIS17; Tripolino, HIS27), two POP accessions (Colosseo, POP4; Hathor, POP8) and one NDS (NDS 2, Maiorcone). The second sub-group comprised mainly modern cultivars: (Aureo, POP1; Claudio, POP2; Duilio, POP6; Dylan, POP7; Iride, POP9; Neolatino, POP10; Normanno, POP11; Simeto, POP14; Svevo, POP15) and also one NDS1 (Khorasan), variety that very noticeably stands out as it is not even a durum wheat, but fits to a different *Triticum turgidum* subspecies (*Triticum turanicum* Jakubz).

### Gliadins

SDS-PAGE gliadin profiles were also used to further describe the samples in this work. The gliadin profiles may be sub-divided into sulphur-rich (S-rich) α-, β-, γ- sub-fractions and a sulphur-poor (S-poor) ω-gliadin sub-fraction as shown in Fig. 5C. The gel images were analyzed in the same way as for glutenin profiling and again a phylogenetic tree was constructed which displayed the presence of 5 clusters (Fig. 5D). The first cluster comprised only POP samples (Aureo, POP1; Claudio, POP2; Duilio, POP6; Dylan, POP7; Iride, POP9; Simeto, POP14; Svevo, POP15; Tirex, POP16), which presented a high degree of similarity among each other (> 0.70 of DICE coefficient).

The second cluster included a mix of both HIS and POP entries (7 and 4 entries respectively). These samples could be further divided into two subgroups, the first made up of POP accessions with the exception of Cappelli (HIS8), and the second containing exclusively HIS samples, among which Biancuccia (HIS3) and Bidì (HIS4).

Third and fourth clusters showed the presence of many HIS samples, with Bufala rossa (HIS6) displaying the most differentiated gliadin profile, as per its DICE coefficient. Colombo (POP3) was the only modern variety present in cluster 3 and 4.

The last cluster (5) on the other hand, seems to be the only one comprising samples belonging to all groups, including most of the Russello samples (HIS20, HIS21, HIS22, HIS24), two of the NDS samples (Korasan, Maiorcone) and some highly differentiated samples like Margherito (HIS16), Perciasacchi (HIS17), Tripolino (HIS27) that present the least similarity to other members of the cluster.

## Discussion

Beleggia and coworkers^29^ in 2009 were the first to study the aroma of durum wheat wholemeal flour. They were followed in 2015 by Starr’s group who studied the variations in volatile compound profiles of bread wheat (*Triticum aestivum* L.) varieties and landraces^33^. To carry on from these initial works, we present for the first time the combined analyses of storage proteins (gliadin, glutenin) and volatile organic compounds by SDS-PAGE and PTR-TOF analysis respectively, of Triticum wheat kernels and wholemeal flour, obtained from 47 landraces and varieties.

The use of the PTR-MS spectra as fingerprints allowed for the identification of 32 compounds that consistently appeared in both the kernel and the wholemeal flour, which were then used to describe the selected wheat samples. Some of those compounds showed quantitative trends common to the kernel and wholemeal flour.

Our results confirm that a sample may be characterized on the basis of its quantitative VOC profile, and this fact is compatible with the hypothesis that the content of volatile compounds might be under genetic control, which was put forward in a previous report on *Triticum aestivum* L.^33^. The results of our experiments show that this could be valid for both modern varieties and local landraces.

As for the modern varieties, Claudio (POP2), Duilio (POP6) and Svevo (POP15) are good examples. They displayed a distinctive VOC profile independent of which matrix was considered. In wholemeal flour, two of the most distinguishing compounds are 2,3-butanedione (C23, C_4_H_6_O_2_) - an important odor-active molecule in fresh rye flour^39^ - and methylsulphanylmethane (C27, C_2_H_5_O_2_), which had previously been found to be present in large quantities in the modern varieties of spring wheat^33^.

Quantitative differences were found in some local landraces which distinguish them from modern varieties. Bidì (HIS4) was used to represent the landraces and it showed a specific VOC profile for both wholemeal flour and kernel: e.g. the presence of high level of dimethyl sulphide (C14, C_2_H_6_S). This compound naturally occurs in many living systems such plants, algae, bacteria^40^ and fungi (truffles)^36^ and its quantitative level is not affected by grinding conditions. In the spring wheat species Maiorcone (NDS2, *Triticum aestivum* L.), some features that distinguish it were detected only for kernel data (Fig. 2A) and no differences were detected for the other members of the NDS group (Khorasan, NDS1, *Triticum turanicum* Jakubz; Romano, NDS3, *Triticum aestivum* L). As to quantitative differences between the matrices, 2-propenal (C10, C_3_H_4_O) and 2-butanone (C17, C_4_H_8_O) displayed higher levels in kernel than in wholemeal flour (Fig. 1) contrary to results in previously published^29^ work in which aldehydes and ketones were reported to increase during kernel grinding as alcohol and ester compounds decrease.

In general, the grinding phase required to obtain wholemeal flour resulted in VOC profiles that showed quantitative differences when compared to kernel profiles. This is true for two classes in particular: aromatic hydrocarbons and sulphur-containing compounds. The first class includes the aromatic hydrocarbons benzene (C20, C_6_H_6_), phenylethene (C28, C_8_H_8_) and 1,3-dimethylbenzene (C29, C_8_H_10_) whereas the second one includes compounds such as diethyl sulphide (C25, C_4_H_10_S). It might be that the disruption of the kernel structure which occurs during grinding results in the release of some compounds.

SIMPER results (Table 4) showed that the contribution of a compound to sample discrimination could change with the matrix (kernel vs wholemeal flour). The pair-wise comparisons A and C, which include wholemeal flour data, shows how compounds with relatively high molecular weights contribute to sample discrimination, whereas for comparisons among kernels only, compounds with low *m/z* are dominant.

The tanglegram (Fig. 4) tells how closely or distantly the varieties are related to each other both when the kernel is used and when the wholemeal flour is used, and also how these relations change when moving from one matrix to the other. By looking at how samples distribute in the phylogenetic trees it is possible to see how similar or different their VOC profiles are, whether or not the modern varieties cluster separately from the local landraces, and what subgroups form. The entanglement value (0.42) confirmed that the grinding phase modifies the existing relationships compared to what they were in the unprocessed kernels. This evidence further supports the idea that a major release of certain VOC can be imputed to the grinding process.

To complement the VOC profiles, the storage proteins glutenin and gliadin were separated by electrophoresis for further analysis. Cluster analysis of the glutenin fraction (Fig. 5B) shows how the samples grouped into four main branches. Distinctive profiles could be observed within modern cultivars as reported by the DICE coefficient. Some examples are Creso (POP5) and Sculptur (POP13). The same is true for some of the local landraces such as Aziziah (HIS1), Cicireddu (HIS9) and Realforte rosso (HIS19).

The cluster analysis of the gliadin fraction (Fig. 5D) divided the samples into five branches. On the whole, modern cultivars grouped separately from local landraces. A notable exception is Sculptur (POP13), which exhibits a gliadin profile unlike other modern cultivars, similarly to what was observed for this cultivar’s glutenin data. Local landraces again exhibited high degrees of dissimilarity, which facilitates their characterization. Two good examples are Bufala rossa (HIS6) and Aziziah (HIS1).

In this work, we have presented the electrophoretic characterization of the storage proteins together with VOC data for 47 durum wheat varieties and landraces. Our data further validates the usefulness of the direct injection mass spectrometry method PTR-TOF as a tool to discriminate among food matrices. One of the more notable advantages of this method is that it allows for the analysis of a large number of samples (high-throughput) and that is clearly demonstrated in this work, as well as in a previous pioneering work^41^. With the help of this tool, we were able to find the 32 compounds listed in this paper, as well as to quantify them precisely enough to be able to individuate significant differences between the two matrices of what substantially was the same material: the kernel (whole) versus the wholemeal flour (the kernel grinded), and as such determine the often subtle differences caused by the mechanical processing. In our samples, the grinding induced a release of, for example, aromatic hydrocarbons and sulphur compounds. From the data gathered here, emerges a clear picture of how different modern cultivars are from local, historical landraces, as proven by the statistical analysis and as previously reported for *Triticum aestivum* L.^33^. The storage protein data consolidates this trend.

In conclusion, this combined approach appears to be a promising method for the full and precise characterization of durum wheat varieties and landraces and it appears as a step forward in the selection of appropriate raw materials to correctly predict and achieve the desired end product flavor, protein characteristics, and texture or technological properties.

## Methods

### PTR-TOF-MS analysis

VOC analysis was carried out on 47 accessions of wheat (Table 1) collected from Azienda Pietranera, the experimental station of the University of Palermo, located in central-southern Sicily, Italy, (37°30′N, 13°31′E). Five accessions from the Russello population were included in the present study, selected from different environmental niches existing in Sicily.

The samples were classified into three groups according to plant height, the spread of varieties and release date (Table 1). Out of the 47 accessions, 17 were modern varieties (hereafter named “POP”), released between 1974 (cv. Creso) and 2010 (cv. Colombo); this group comprises the most spread durum wheat varieties grown today in Southern Italy and in other areas of the Mediterranean basin. Twenty-seven were ancient landraces and varieties (hereafter named ancient genotypes, “HIS”), widely grown at the end of the XIX and in the first half of the XX century in Southern Italy and particularly in Sicily. Some of these accessions (such as Perciasacchi, Cappelli, Russello, Bidì) are today object of a renewed interest by both farmers and consumers who ascribe to their distinctive sensorial and nutritional properties. The remaining three accessions, Khorasan (*Triticum turanicum* Jakubz) and the two landraces, Maiorcone and Romano (*T. aestivum L.*), were selected as outlier samples belonging to different *Triticum* species (hereafter named not durum species, “NDS”).

Analyses were carried out on whole kernels and also on wholemeal flour from mechanical grinding. Briefly, 15 ± 0.50 grams of kernels were collected from each sample, heated to 60 °C for 15 minutes and then used for the first step of the analysis. Next, samples were immediately ground in a lab mill, to a particle size of <200 μm. VOCs were then measured in the resulting wholemeal flour after a further heating step.

Volatiles were analyzed with a PTR-TOF 8000 (Ionicon Analytik GmbH, Innsbruck, Austria) using H_3_O^+^ as reagent ion for the proton transfer reaction. The H_3_O^+^ ions react with all the biogenic VOCs having a proton affinity higher than that of water (165.2 kcal mol^−1^). The single ion products of this reaction then separate in the reaction chamber (Drift tube) based on their mass to charge (*m/z*) ratio under controlled conditions: applied voltage set at 600 V, temperature 110 °C, and pressure 2.25 mbar.

Compounds such as 1,4-dichlorobenzene (*m/z* = 146.976) and 1,2,3-trichlorobenzene (*m/z* = 180.937) were continuously used, together with other known low mass ions, for a precise conversion of ‘time-of-flight‘ into ‘mass-to-charge‘ ratio (*m/z*) in order to calibrate the mass scale and the sum formula of the unknown ions^42^,^43^. For the analysis, each sample was placed in a glass jar and covered with a special lid that containing a Teflon connection to a zero-air generator (inlet) and to the PTR-TOF-MS system (outlet). Analyses were carried out 5 times on each sample. The headspace was then measured by direct injection into the PTR-TOF-MS drift tube inlet for 180 seconds, after 120 seconds of exposure. Preliminary measurements on empty jars were run before every experiment and used for background subtraction. All mass spectra up to *m/z* = 250 were simultaneously detected and recorded with 1 s as the integration time. Internal calibration was based on *m/z* = 21.0202 (H_3_^18^O^+^), *m*/*z* = 59.0491 (C_3_H_6_O^+^) and *m/z* = 181.937 (C_6_H_4_Cl_3_^+^). Further information was obtained from literature^42^,^43^. Raw spectra acquisition and peak quantification were performed as described in reference^36^.

External calibration was automatically done by the acquisition program and it achieved a mass accuracy of 0.0001 Th (1 Thomson = 1 Da/e) for the selected mass range^36^. Peak data were extracted and then quantified as pptv (parts per trillion by volume).

### Gliadin and Glutenin protein analysis

#### Protein Extraction

Protein analyses were carried out on fractions linked to gluten formation. Gliadin and glutenins were extracted using a protocol from Singh *et al*.^44^ with modifications as follows: Proteins were extracted from ground kernel samples. About 100 mg of wholemeal flour were used by adding 0.75 ml of 1-propanol 50% solution). Samples were then heated for 30 min at 65 °C in a thermoshaker (1,400 rpm) and centrifuged at 10,000 rpm for 2 minutes. This procedure was done twice and, the resulting pellet was used as starting material from which to extract glutenins (a) whereas the supernatant was used for gliadins (b) extraction:

- Glutenins: 1.5% solution (2% DTT, DL-dithiothreitol) for the reduction of disulfide bridges was added to the pellet and the pellet was then transferred to a thermomixer at 65 °C for 30 min (1,400 rpm). After a centrifugation step (2 min at 10,000 rpm) in which the pellet was recuperated, 0.1 ml of 1.4% of alkylating solution (4-vinylpyridine) was added to extract followed by a new centrifugation step at 13,000 rpm for 5 minutes. Subsequently, 180 μl of supernatant were mixed with 180 μl of extraction solution A (1:1 ratio) (pH 6.8 (2% SDS (w/v), 40% glycerol (w/v), 0.02% (w/v) bromophenol blue)) and the mixture thus obtained was placed in a thermoshaker at 90 °C for 5 min (1,400 rpm). After that, product was centrifuged and 10 μl of the supernatant were used for electrophoretic analysis.
- Gliadins: the extraction of gliadin fraction was carried out on the supernatant obtained as described above, heated to 65 °C for 5 hours to eliminate the alcoholic fraction, then mixed with 0.3 ml of extraction solution B (pH 8.0, 2% SDS (w/v), 40% glycerol (w/v), 0.02% (w/v) bromophenol blue)). The extract was subsequently transferred to a thermomixer for 5 minutes of shaking at 90 °C (1,400 rpm) and then centrifuged at 10,000 rpm for 5 min. 10 μl of the supernatant were used for electrophoretic analysis.

#### SDS-PAGE and bioinformatics analysis

Proteins were separated using the well-established technique of SDS-PAGE. In this case, the gels used measured 20cm by 20 cm, had a thickness of 1 mm and consisted in a stacking gel (4.8% T, 1.3% C, pH 6.8) and a running gel (15%, 1.3% C, pH 8.5). Each sample was run 4 times (*n* = 4). Electrophoresis runs were carried out using the Protean XI cells (Bio-Rad Laboratories, Inc, Hercules CA) with the following parameters: 25 mA for each gel, running time 8 hours, temperature 15 °C. Precision Plus unstained (#161-0363, Bio-Rad Laboratories, Inc, Hercules CA) was used as molecular marker.

After electrophoretic separation, the gels were stained using a 12% solution of TCA (trichloroacetic acid) for 5 minutes, followed by a solution of 0.1% Coomassie R in methanol/acetic acid (ratio 4:1) for 4 hours.

Destaining took place in MilliQ water (Millipore Corp., Bedford, MA) for 12 hours at room temperature. The gel images were acquired using a Bio-Rad densitometer GS-800™ in greyscale, 300 dpi. The images were then analyzed using the “Quantity One 1-D Analysis™” software (Bio-Rad Laboratories, Inc, Hercules CA).

### Statistical analysis

To identify inter-specific and intra-specific relationships related to VOCs and proteomic profiles of *Triticum* spp. accessions, samples were classified into three groups of data (HIS, POP and NDS). Quantitative data were depicted as a heat map to display the complex data in matrix format. A heat map does two things on a data matrix. First, rows and columns could be rearranged so that the rows (or columns) that present similar profiles be grouped, making them easier to visualize. Second, every item in the data array is displayed as a color, making it possible to graphically display the pattern. A dendrogram based on signal quantities was created using correlation-based distances and the Ward’s method of agglomeration^38^.

Data from PTR-TOF-MS were also analyzed using a PCA (principal component analysis) for both wholemeal flour and kernel samples and their results were graphically processed to highlight the contribution of each variable respectively (identified compounds) in the samples differentiation.

A tanglegram was generated to illustrate the similarities and divergences the associations and putative codivergence between the two dendrograms, using the Ward’s method for hierarchical cluster analysis^38^. The rooted phylogenetic trees (wholemeal flour and kernel) are drawn opposite each other, using auxiliary lines to connect samples and establish a network of interactions.

All computations were performed with R 3.2.3 language and environment^45^ and additional packages circlize^46^, dendextend^47^ and FactoMineR^48^.

Three accessions (both kernel and wholemeal flour) were chosen based on PCA results as representative of each of three groups (HIS, POP, NDS). To further examine the differences between the genotypes and treatment, the percentage contribution of each compound to the average dissimilarity between the aforementioned factors was calculated with similarity percentage analysis (SIMPER^49^). Compounds that accounted for 70% of the cumulative percent contribution (**∑δ**_**i**_**%**) of the total dissimilarity were identified and then ranked in increasing order of contribution.

Protein data from SDS-PAGE were compared through the similarity matrix that contains the similarity values of all the lanes in a gel image. Lane similarity was computed using the DICE coefficient^50^. The dice similarity coefficient (DSC) is a spatial overlap index and a reproducibility validation metric^51^. The value of a DSC ranges from 0, indicating no spatial overlap between two sets of binary segmentation results, to 1, indicating complete overlap. Results were then displayed as phylogenetic tree computed using the Ward’s method^38^ as cluster method.

## Additional Information

**How to cite this article**: Vita, F. *et al*. Aromatic and proteomic analyses corroborate the distinction between Mediterranean landraces and modern varieties of durum wheat. *Sci. Rep.* **6**, 34619; doi: 10.1038/srep34619 (2016).

## Figures and Tables

**Table 1:** List of wheat varieties and landraces selected for VOC and protein analyses.

Group	Entry	Year of release	Pedigree
HIS1	AZIZIAH	1920–1925	North-African selection from Palestinian landraces
HIS2	BIANCOLILLA	<1915	Indigenous landrace from Sicily
HIS3	BIANCUCCIA	<1915	Indigenous landrace from Sicily
HIS4	BIDÌ	<1915	Indigenous landrace from Sicily
HIS5	BUFALA NERA	<1915	Indigenous landrace from Sicily
HIS6	BUFALA ROSSA	<1915	Indigenous landrace from Sicily
HIS7	CAPEITI	1950	Eiti 6 × Cappelli
HIS8	CAPPELLI	1915	Selection from North-African landrace “Jean Rhetifah”
HIS9	CICIREDDU	<1915	Indigenous landrace from Sicily
HIS10	DAUNO	1930–1940	Selection by Strampelli from materials of unknown origin
HIS11	DAUNO III	1930–1940	Selection by Strampelli from materials of unknown origin
HIS12	GARIGLIANO	1926	Tripolino × Cappelli
HIS13	GIGANTE	<1915	Indigenous landrace from Sicily
HIS14	GRIFONI 235	1949	Cappelli × Triticum aestivum
HIS15	MADONIE	<1915	Indigenous landrace from Sicily
HIS16	MARGHERITO	<1915	Indigenous landrace from Sicily
HIS17	PERCIASACCHI	<1915	Indigenous landrace from Sicily
HIS18	REALFORTE BIANCO	<1915	Indigenous landrace from Sicily
HIS19	REALFORTE ROSSO	<1915	Indigenous landrace from Sicily
HIS20	RUSSELLO 1	<1915	Indigenous landrace from Sicily
HIS21	RUSSELLO 2	<1915	Indigenous landrace from Sicily
HIS22	RUSSELLO 3	<1915	Indigenous landrace from Sicily
HIS23	RUSSELLO 4	<1915	Indigenous landrace from Sicily
HIS24	RUSSELLO 5	<1915	Indigenous landrace from Sicily
HIS25	SCORSONERA	<1915	Indigenous landrace from Sicily
HIS26	SICILIA	<1915	Indigenous landrace from Sicily
HIS27	TRIPOLINO	1920–1925	North-African selection from Palestinian landraces
POP1	AUREO	2009	Kofa × Svevo
POP2	CLAUDIO	1998	(Cimmyt selection × Durango) × (IS193B × Grazia)
POP3	COLOMBO	2010	Biensur × Nefer
POP4	COLOSSEO	1995	Creso × Mexa (Mutant)
POP5	CRESO	1974	Cpb144 × [(Yt54-N10-B)Cp2 63 Te3]
POP6	DUILIO	1984	Cappelli × (Anhinga × Flamingo)
POP7	DYLAN	2002	Neudur × Ulisse
POP8	HATHOR	2006	FD495 × Khorasan
POP9	IRIDE	1996	Altar 84 × Ares sib
POP10	NEOLATINO	2005	(Latino × Trinakria) × MG1433
POP11	NORMANNO	2002	(Simeto × F22) × L35
POP12	SAN CARLO	1996	Grazia × Degamit
POP13	SCULPTUR	2007	not available
POP14	SIMETO	1988	Capeiti 8 × Valnova
POP15	SVEVO	1996	Cimmyt selection × Zenit
POP16	TIREX	2007	Svevo × Nefer
POP17	VALBELICE	1992	O111 × BC5
NDS1	KHORASAN^#^	<1915	Indigenous landrace from “Near East”
NDS2	MAIORCONE*	<1915	Indigenous landrace from Sicily
NDS3	ROMANO*	<1915	Indigenous landrace from Sicily

Varieties have been classified in three groups (HIS, POP, NDS). All entries are *Triticum durum* Desf., except: ^#^*****Triticum turanicum* Jakubz, ^*^Triticum aestivum L. THe majority of pedigree information was provided from Genetic Resources Information System for Wheat and Triticale (GRIS) (last update: 2016-03-24).

**Table 2:** Compounds tentatively identified through PTR-Analysis.

#^a^	Protonated theoretical *m /z*^b^	Protonated chemical formula^c^	Tentative identification^d^	Reference^e^
C1	27.022	C_2_H_3_^+^	**Acetylene**	36
C2	33.033	CH_5_O^+^	**Methanol**	52, 53, 54
C3	39.020	C_3_H_3_^+^	**Isoprene fragment**	55
C4	41.038	C_3_H_5_^+^	**Alkyl fragment**	36, 52
C5	43.018	C_2_H_3_O^+^	**Alkyl fragment**	n. d.
C6	43.050	C_3_H_7_^+^	**Alkyl fragment**	52
C7	45.033	C_2_H_5_O^+^	**Acetaldehyde**	43, 52, 53, 56
C8	47.049	C_2_H_7_O^+^	**Ethanol**	52, 53, 56
C9	49.010	CH_5_S^+^	**Methanethiol**	36
C10	57.033	C_3_H_5_O^+^	**2-Propenal (acrolein)**	36, 43
C11	57.070	C_4_H_9_^+^	**Alkyl fragment (hexanol)**	57
C12	59.049	C_3_H_7_O^+^	**Acetone**	33, 52, 53, 58
C13	61.028	C_2_H_5_O_2_^+^	**Acetates**	52, 53
C14	63.027	C_2_H_7_S+	**Dimethyl sulphide**	n. d.
C15	69.069	C_5_H_9_^+^	**Isoprene**	52, 54, 58, 59
C16	71.049	C_4_H_7_O^+^	**2-Butenal**	36
C17	73.064	C_4_H_9_O^+^	**2-Butanone**	52, 56, 57, 60
C18	75.043	C_3_H_7_O_2_^+^	**Methyl acetate**	53, 60
C19	77.041	C_3_H_9_S+	**Propanethiol**	n. d.
C20	79.054	C_6_H_7_^+^	**Benzene**	58
C21	81.069	C_6_H_9_^+^	**Terpene fragment**	53, 57
C22	83.085	C_6_H_11_^+^	**C6 fragment (hexenals/hexenols)**	43
C23	87.045	C_4_H_7_O_2_^+^	**2,3-Butanedione (diacetyl)**	53, 60
C24	89.040	C_4_H_9_O_2_^+^	**Methyl propanoate**	60
C25	91.054	C_4_H_11_S^+^	**Diethyl sulphide**	60
C26	93.069	C_7_H_9_^+^	**Toluene**	29, 52, 55, 56
C27	95.020	C_2_H_6_O_2_S^+^	**Methylsulphanylmethane**	33
C28	105.069	C_8_H_9_^+^	**Phenylethene (styrene)**	60
C29	107.085	C_8_H_11_^+^	**1,3-Dimethylbenzene (xylene)**	29, 58
C30	109.078	C_6_H_9_N_2_^+^	**2,3-Dimethylpyrazine**	33
C31	120.100	C_8_H_9_O^+^	**Acetophenone**	33, 59
C32	123.080	C_8_H_11_O^+^	**2-Phenylethyl alcohol**	n. d.

^**a**^Unique code assigned to each compound. ^**b**^Theoretical mass to charge ratio (*m/z*) found in literature or in PTR-TOF-MS manual. ^**c**^Compound’s protonated chemical formula. ^**d**^Tentative identification based on spectral properties. ^e^Bibliographical reference. Compounds were reported in the text with non-protonated formula.

**Table 3:** Compounds significantly correlated to first and second dimensions of the principal component analysis (PCA) for kernel and wholemeal flour data.

Kernel				Wholemeal flour			
First dimension (PC1)		Second dimension (PC2)		First dimension (PC1)		Second dimension (PC2)	
Code^a^	*r*^b^	Code^a^	*r*^b^	Code^a^	*r*^b^	Code^a^	*r*^b^
**C2**	*0,893*	**C7**	*0,728*	**C7**	*0,811*	**C18**	*0,846*
**C32**	*0,746*	**C30**	*0,694*	**C32**	*0,775*	**C23**	*0,824*
**C31**	*0,746*	**C22**	*0,685*	**C1**	*0,760*	**C13**	*0,813*
**C30**	*0,650*	**C31**	*0,628*	**C8**	*0,726*	**C21**	*0,797*
**C17**	*0,640*	**C32**	*0,627*	**C3**	*0,663*	**C5**	*0,694*
**C8**	*0,619*	**C1**	*0,573*	**C6**	*0,644*	**C24**	*0,692*
**C12**	*0,613*	**C5**	*0,563*	**C4**	*0,638*	**C27**	*0,640*
**C4**	*0,601*	**C23**	*0,442*	**C17**	*0,636*	**C16**	*0,492*
**C3**	*0,544*	**C21**	*0,368*	**C31**	*0,634*	**C30**	*0,434*
**C9**	*0,328*	**C15**	*0,365*	**C15**	*0,618*	**C10**	*−0,328*
**C11**	*0,302*	**C13**	*0,308*	**C30**	*0,575*	**C2**	*−0,375*
**C24**	*−0,288*	**C16**	*−0,380*	**C5**	*0,430*	**C3**	*−0,387*
**C29**	*−0,313*	**C14**	*−0,386*	**C22**	*0,378*	**C17**	*−0,476*
**C5**	*−0,336*	**C10**	*−0,386*	**C16**	*0,350*	**C12**	*−0,557*
**C13**	*−0,376*	**C4**	*−0,515*	**C2**	*0,333*	**C4**	*−0,667*
**C25**	*−0,439*	**C11**	*−0,529*	**C14**	*−0,324*	**C11**	*−0,765*
**C23**	*−0,442*	**C6**	*−0,549*	**C25**	*−0,900*		
**C19**	*−0,479*			**C19**	*−0,923*		
**C26**	*−0,487*			**C28**	*−0,966*		
**C28**	*−0,590*			**C29**	*−0,966*		
**C27**	*−0,594*			**C20**	*−0,967*		
**C21**	*−0,657*						
**C20**	*−0,715*						

The selection of significant compounds was done based on their correlation coefficients. (α = 0.05) and sorted by Pearson correlation coefficient. ^a^Unique code assigned to each compound. ^b^Pearson’s correlation coefficient.

**Table 4:** SIMPER results of pair-wise comparisons on selected samples.

A					
Pair-wise Comparisons: Kernel vs Wholemeal Flour					
HIS5 vs HIS5		NDS1 vs NDS1		POP2 vs POP2	
Code^a^	∑δi%^b^	Code^a^	∑δi%^b^	Code^a^	∑δi%^b^
C12	0,120	C25	0,121	C1	0,087
C25	0,240	C20	0,232	C3	0,162
C20	0,336	C12	0,324	C4	0,230
C3	0,431	C29	0,409	C6	0,298
C29	0,521	C2	0,486	C25	0,364
C28	0,584	C3	0,558	C7	0,431
C8	0,639	C8	0,623	C2	0,492
C4	0,692	C17	0,682	C13	0,543
C6	0,736	C28	0,722	C10	0,585
				C8	0,628
				C27	0,666
				C21	0,704
**B**					
**Pair-wise Comparisons: Kernel vs Kernel**					
**HIS5 vs NDS1**		**HIS5 vs POP2**		**NDS1 vs POP2**	
Code^a^	∑δi%^b^	Code^a^	∑δi%^b^	Code^a^	∑δi%^b^
C14	0,155	C2	0,086	C12	0,103
C3	0,247	C6	0,165	C2	0,195
C6	0,331	C14	0,239	C1	0,273
C12	0,403	C7	0,308	C7	0,350
C4	0,469	C12	0,372	C3	0,409
C17	0,534	C1	0,434	C4	0,462
C1	0,590	C8	0,491	C6	0,514
C8	0,645	C4	0,545	C25	0,564
C7	0,694	C25	0,599	C21	0,608
C2	0,726	C21	0,647	C8	0,650
		C23	0,686	C17	0,691
		C5	0,724	C5	0,731
**C**					
**Pair-wise Comparisons: Wholemeal Flour vs Wholemeal Flour**					
**HIS5 vs NDS1**		**HIS5 vs POP2**		**NDS1 vs POP2**	
Code^a^	∑δi%^b^	Code^a^	∑δi%^b^	Code^a^	∑δi%^b^
C12	0,142	C29	0,069	C1	0,093
C3	0,280	C25	0,137	C13	0,186
C14	0,378	C20	0,205	C3	0,266
C1	0,456	C12	0,273	C4	0,342
C4	0,509	C21	0,337	C21	0,414
C25	0,561	C1	0,396	C7	0,471
C29	0,606	C14	0,454	C20	0,518
C28	0,648	C13	0,508	C2	0,562
C13	0,690	C28	0,56	C29	0,605
C20	0,728	C27	0,606	C6	0,648
		C2	0,652	C25	0,684
		C4	0,690	C12	0,717
		C23	0,722		

(**A**) Representation of the pair-wise comparison wholemeal flour *vs* kernel (**B**) Representation of the pair-wise comparison kernel vs kernel (**C**) Representation of the pair-wise comparison wholemeal flour vs wholemeal flour. HIS5 = Bidì, NDS1 = Khorasan, POP2 = Claudio. ^**a**^Compound code as reported in Table 2. ^**b**^Cumulative contribution percentage.
